# Editorial: Fungal wheat diseases: etiology, breeding, and integrated management, volume II

**DOI:** 10.3389/fpls.2023.1247327

**Published:** 2023-07-25

**Authors:** María Rosa Simón, Paul C. Struik, Andreas Börner

**Affiliations:** ^1^ Cerealicultura, Facultad de Ciencias Agrarias y Forestales, Universidad Nacional de La Plata, La Plata, Argentina; ^2^ Department of Agricultural and Forestry Technology, Laboratory of Extensive Agricultural Production (LAPROAGRE), Consejo Nacional de Investigaciones Científicas y Técnicas La Plata (CONICET), La Plata, Argentina; ^3^ Department of Plant Sciences, Centre for Crop System Analysis, Wageningen University & Research, Wageningen, Netherlands; ^4^ Genebank Department, Leibniz Institute of Plant Genetics and Crop Plant Research, Gatersleben, Germany

**Keywords:** impact of wheat diseases, pathogen identification, infection structures, resistance of isolates to stresses, wheat defense mechanisms, integrated management

## Relevance of wheat and wheat diseases

Wheat is the most widely grown crop in the world in terms of area and is the major source of calories and plant-derived protein in human food. Advances in wheat yield rather than land expansion have been the main drivers of the steady growth of global wheat production (Erenstein et al., 2022), which is currently at 800 million tons (FAO, 2023). Wheat production is continuously threatened by factors such as a lack of suitable farmland, climate change and abiotic and biotic stressors. Future demand must be met by combining integrated disease and pest management, adaptation to warmer climates and abiotic stresses, and sparing use of water and other resources. A storm of newly emerging pathogens, caused by the loss of wheat’s genetic diversity and the search for high-performing cultivars, has made diseases a serious concern (Figueroa et al., 2018).

In a first Research Topic on fungal wheat diseases, we brought together 26 papers, including reviews, original research papers and a methodological papers (Simón et al., 2021). This second volume on fungal wheat diseases consists of two reviews and 10 original papers. Advances on the probabilistic impact and identification of the main pathogens, functions of infection structures, the key infection stages of defense in high-temperature-resistant isolates, as well as metabolic changes in response to wheat pathogens are addressed. Aspects essential for proper integrated management, such as models to predict the risk infection index, mechanisms of seed transmission, genetics and breeding for resistance, and biological control are also considered.

## Impact of wheat diseases

Pests and diseases are responsible for 21.5% of the current crop losses (Savary et al., 2019). Fungal diseases such as leaf and stripe rust, Fusarium head blight, Septoria leaf blotch, spot blotch, tan spot and powdery mildew cause the most significant losses out of the 31 pests and pathogens reported in wheat (Savary et al., 2019).

In this Research Topic, Chai et al. examined the probabilistic impact of five major fungal pathogens of wheat (leaf, stripe and stem rust, Septoria tritici blotch and Fusarium head blight) on wheat production. They determined that almost 90% of the wheat area is at risk from at least one of these diseases, resulting in annual losses of more than 62 million tons, and that more research on the subject is economically justified.

## Identification of pathogens in wheat, infection structures and mechanisms of resistance of isolates to environmental stresses

To predict outbreaks, accurate pathogen identification is crucial. Fungal diseases can spread by releasing spores from infected plants or stubble (Suffert et al., 2011). Pilo et al. identified 150 species of fungal pathogens of cereals, using microscopy and two metagenomic-based methods useful for the forecasting of disease outbreaks without knowledge about which pathogens are present. They also found that some of the pathogens’ spore release is correlated with environmental variables. Such correlations can be used to manage the diseases.

Fungal pathogens possess different infection structures. The haustorium is an infection structure present in biotrophic pathogens and is a key player in the establishment of pathogenesis (Jaswal et al., 2020). Mapuranga et al. assessed the state-of-the-art on the establishment and development of the haustorium, its structure, composition, gene expression, mode of nutrient acquisition and the haustorial effector secreted into the host cell, including the recent haustorial transcriptome investigations.

Plant pathogens have evolved the capacity to adapt to changing environments in order to survive (Knies and Kingsolver, 2010). Zhang et al. found that haustoria formation and hyphae expansion were the key infection stages when comparing high-temperature resistant with sensitive isolates of *Blumeria graminis* f.sp. *tritici.* Additionally, they discovered that high-temperature-resistance is related to the induction of heat shock protein genes in response to stress at those stages.

## Defense mechanisms of wheat against pathogens

Numerous substances (from cell wall components to metabolic enzymes) that are crucial to the molecular and cellular signaling processes during pathogen infection, have been documented to shield plants from pathogen invasion and provide hosts with induced resistance (Kaur et al., 2022). Maserumule et al. analyzed the biochemical alterations in wheat metabolite expression in response to stem rust infection. They discovered biomarkers such as fatty and carboxylic acids, various sugars and phenolic chemicals, including flavonoids, hydrocinnamic acid derivatives, and polyphenols, linked to the interaction between wheat and *Puccinia graminis*. The most significant metabolic pathways linked to the metabolites regulating wheat defense were those involving riboflavin, cutin and suberin, thiamine, folate, and alpha-linolenic acid, as well as glyoxylate and dicarboxylate metabolism.

## Integrated management of wheat diseases

Epidemic forecasting is relevant for effective integrated management strategies. Understanding how weather and heading dates affect diseases is crucial to preventing many epidemics (Simón et al., 2005). Jung et al. modeled prospective Fusarium head blight (FHB) epidemics in Korea using weather conditions during flowering. They showed that epidemics would gradually worsen and that the early-heading wheat cultivars would show decreases in FHB epidemics, highlighting the significance of taking adaptation measures to prevent a rise of the disease as a result of climate change.

Verifying the presence of pathogens in the seeds and their transmission to plants is crucial in the context of integrated management. Surovy et al. did not find evidence of systemic transfer of *Magnaporthe oryzae* pathotype *Triticum* (MoT), the causal agent of wheat blast, from infected seed to seedlings after tillering. However, the presence of MoT in seedlings may act as a source of inoculum, which might contaminate seeds by air-borne infection. Results highlight the dangers of the spread of wheat blast across continents through seeds and the significance of using healthy seeds.

Acceptable levels of resistance, adequate fertilization management, crop rotation, proper timing of planting and tillage that enhance residue decomposition, complemented with biological and/or chemical control, are also relevant strategies that can help minimize the severity of diseases (Simón et al., 2020).

## Genetic resistance to wheat diseases

In this Research Topic, Gupta et al. reviewed the resistance to four relevant wheat pathogens causing Septoria nodorum blotch, tan spot, spot blotch and Septoria tritici blotch with emphasis on the necrotrophic effectors and sensitivity genes in the inverse gene for gene model. This contribution also includes recent information on the whole-genome sequences of the four pathogens.

Nyamesorto et al. identified and edited wheat genes that aid rust pathogens during infection to provide wheat with passive resistance to rust. They identified a wheat transcription factor as a rust pathogen target. By altering it, the cultivars Chinese Spring and Cadenza became more resistant to stem rust and some races of the stem and stripe rust pathogens, respectively, and novel germplasm of wheat was also created.

Ma et al. summarized the genes conferring FHB resistance and mycotoxin detoxication discovered in common wheat and its relatives by using forward and reverse genetic approaches. They also discussed the effects of several resistance genes and the role of host induced gene silencing in enhancing the resistance to FHB as well as the molecular basis of the resistance and the use of the cloned genes for FHB management.

Singh et al. analyzed the genetic architecture of resistance to spot blotch in wheat using a genome-wide association study (GWAS) including 303 genotypes genotyped with 12,196 SNPs. They found 306 marker trait associations (MTAs), some of them significant with the five GWASs used, which were utilized for finding candidate genes. Seven MTAs were considered relevant for marker-assisted selection.

## Biological control for wheat diseases

Biological control is a sustainable method for managing diseases. Plant growth-promoting rhizobacteria increase the control of soil-borne diseases; however, their effect on pathogenic fungi or arbuscular mycorrhizal fungi (AMF) is unclear. Ji et al. found that inoculation with some *Bacillus* strains enhanced rhizosphere soil chemical characteristics and wheat yield as well as lowered the incidence of diseases, rhizosphere fungus, and AMF fungal diversity.

## Conclusion

This second volume on wheat fungal diseases creates an interesting supplement to the first Research Topic. The papers in this second volume highlight the rapid progress that can be made in knowledge with the latest research techniques, progress that is much needed to combat the ever-increasing threats caused by fungal diseases in wheat production across the globe.

## Author contributions

MS prepared the draft. PS and AB revised the draft and approved the final submission. All authors contributed to the article and approved the final version.
